# FAK-targeting PROTAC as a chemical tool for the investigation of non-enzymatic FAK function in mice

**DOI:** 10.1007/s13238-020-00732-8

**Published:** 2020-05-25

**Authors:** Hongying Gao, Chunwei Zheng, Jian Du, Yue Wu, Yonghui Sun, Chunsheng Han, Kehkooi Kee, Yu Rao

**Affiliations:** 1 MOE Key Laboratory of Protein Sciences, School of Pharmaceutical Sciences, MOE Key Laboratory of Bioorganic Phosphorus Chemistry & Chemical Biology, Tsinghua University, Beijing, 100084 China; 2 Tsinghua University-Peking University Joint Center for Life Sciences, Beijing, 100084 China; 3 State Key Laboratory of Stem Cell and Reproductive Biology, Institute of Zoology, Chinese Academy of Sciences, Beijing, 100084 China; 4 Center for Stem Cell Biology and Regenerative Medicine, Department of Basic Medical Sciences, School of Medicine, Tsinghua University, Beijing, 100084 China; 5 University of Chinese Academy of Sciences, Savaid Medical School, Beijing, 100049 China

**Dear Editor**,

Animal models, most commonly mice, that lack a protein of interest play an important role in phenotypic and functional studies of a target gene, allowing researchers to answer various biological questions (Chaible et al., 2010). At present, a variety of tools act at the DNA or RNA level to enable researchers to model gene function (and thus protein) deficiency, including nucleic acid-based RNA interference (Elbashir et al., 2001), antisense oligonucleotides (Schoch and Miller, 2017), and genome editing-based CRISPR-Cas9 (Doudna and Charpentier, 2014) strategies. However, challenges remain. RNA and DNA-based technologies lack exquisite temporal control of the target gene at specified time points in an organism’s development, and they fail to realize acute and reversible target gene function (Chan, 2013). These shortcomings have garnered widespread concern in both fundamental research and drug development. Furthermore, gene knockout will often lead to embryonic lethality, precluding the study of post-embryonic pathophysiological functions of target genes and proteins of interest (Dhanjal et al., 2017).

Proteolysis targeting chimera (PROTAC) is a novel chemical knockdown technology for the post-translational study of proteins of interest. PROTACs are hetero-bifunctional small molecules, which can drive E3 ubiquitin ligase to bind with the target protein, resulting in ubiquitination of the target protein and consequent proteasome-mediated degradation (Raina and Crews, 2010) (Fig. 1A). Unlike classic inhibitors, PROTAC eliminates rather than inhibits both enzymatic and non-enzymatic protein functions. Furthermore, unlike nucleic acid (e.g., siRNA) and genome editing-based (e.g., CRISPR-Cas9) strategies (Cong et al., 2013; Deng et al., 2014), the small molecule-based PROTAC approach is capable of degrading target proteins without requiring any genetic manipulation, guaranteeing the integrity and stability of the genome, which especially suitable for knockdown of embryonic lethal protein. Thus, PROTACs offer significantly broader therapeutic applicability than DNA or RNA-targeting strategies for protein knockdown *in vivo*.

**Figure 1:**
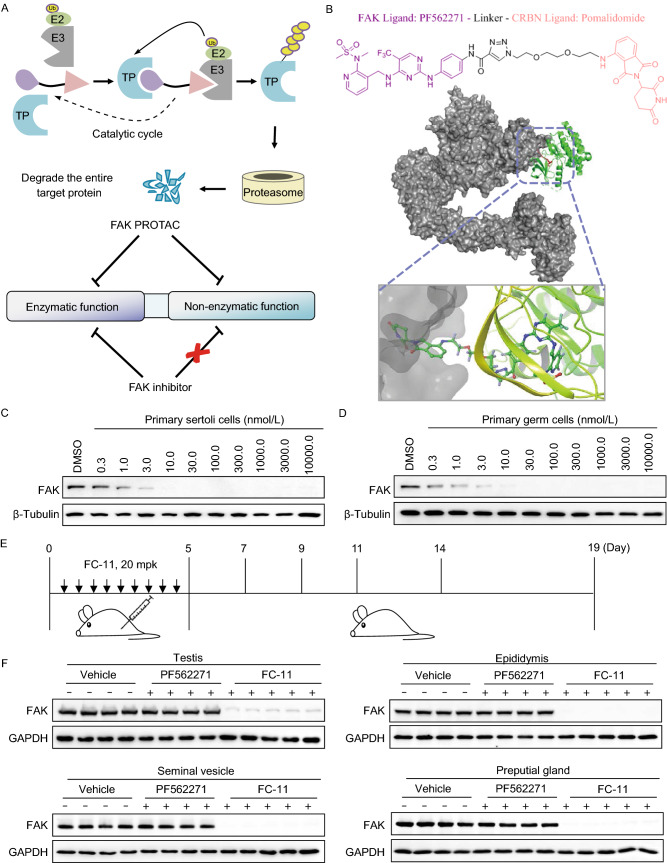

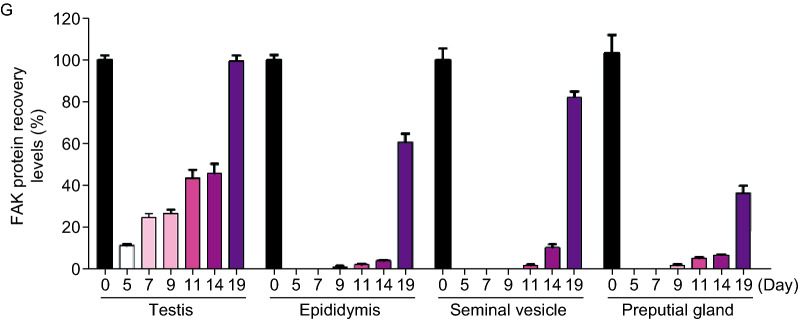
**Rapid and reversible FAK knockdown by FAK targeting PROTAC in male mice reproductive organs**. (A) Schematic depiction of the PROTAC strategy. FAK-PROTAC tool can act on both enzymatic and non-enzymatic functions of FAK, while FAK inhibitor only act on the enzymatic function of FAK. TP, Target Protein. (B) Chemical structure of FAK PROTAC, as shown in the upper portion. Binding mode of PROTAC (ball stick), FAK (PDB 5TOB, green) and CRL4-CRBN (PDB 2HYE and 4CI3, gray) was simulated by Pymol. (C and D) FAK protein degradation in mice primary Sertoli cells and primary Germ cells, the cells were treated at the indicated doses of FC-11 for 8 h. (E) Schematic depiction of FC-11 treated mice for FAK degradation and recovery. (F) FC-11 leads to more extensive FAK degradation in testis, epididymis, seminal vesicle and preputial gland, respectively. Each lane represented a single mouse (*n* = 4 or 5). (G) The recovery ability of FAK in testis, epididymis, seminal vesicle and preputial gland in the indicated days after withdraw administration (*n* = 6). All western blots are the representatives from at least 3 experiments

Focal adhesion kinase (FAK), an embryonic lethal protein, exerts kinase-dependent enzymatic functions and kinase-independent scaffolding functions (Hall et al., 2011). Both functions are crucial in reproduction and early embryonic development (Gungor-Ordueri et al., 2014). Many essential non-enzymatic functions of FAK cannot be investigated with reported FAK kinase inhibitors. To the best of our knowledge, the PROTAC strategy has not been used to study the non-enzymatic function of FAK *in vivo*. It is also unknown whether FAK PROTACs will yield different phenotypes or reveal different FAK functions than kinase-dependent FAK inhibitors *in vivo*. For these reasons, we have chosen FAK as a target to demonstrate the potential utility of the PROTAC strategy for the study of non-enzymatic protein function in mouse reproductive system *in vivo*.

Based on the previous studies of our laboratory, we synthesised the FAK-targeting PROTAC library with FAK ligand of PF562271, cereblon (CRBN)-based E3 ubiquitin ligase ligand of thalidomide, and a variable length of polyethylene glycol or alkyl linkers (Fig. S1). Firstly, we screened the degradation effect of FAK targeting PROTAC molecules in mice primary reproductive related cells. We separated and purified testis-related cells, including primary Sertoli cells and primary germ cells, from 6 dpp C57BL/6N mice, and tested whether degradation resulted from FAK PROTAC library molecules in these primary cells. A remarkable degradation effect, with a DC_50_ of 1.3 nmol/L for primary Sertoli cells and 0.4 nmol/L for primary germ cells, was observed from FAK-PROTAC library molecules, which we confirmed it is FC-11, a PROTAC from our previous reported work (Gao et al., 2019) (Fig. 1B–D). The optimized synthesis route of FC-11 was shown in Supplementary Materials (Scheme 1).

Next, in order to overcome the defect of FAK knockdown *in vivo* caused by existing genetic tools and to clarify the effect of PROTAC tools on the non-enzymatic function of protein in the mouse reproductive system, a few critical issues need to be addressed: 1. Can FC-11 degrade FAK *in vivo*? 2. If it can, is there any different phenotypes between FAK PROTAC and FAK inhibitor? 3. Is the FAK protein degradation reversible?

Encouraged by the results from primary cells, we continued to test FC-11 induced FAK degradation in the reproductive tissues (testis, epididymis, seminal vesicle and preputial gland) of male mice *in vivo* (Fig. S2). Ten-week-old male C57BL/6N mice were administered intraperitoneal injections of FC-11 (20 mg/kg, twice daily [BID]), PF562271 (10 mg/kg, BID), or vehicle control over a 5 day period (Fig. 1E). After 5 days treatment, all FC-11 treated mice exhibited a more than 90% reduction of FAK and phosphor FAK^tyr397^ in the tested reproductive tissues, while PF562271 had no effect on the level of FAK protein, but had an inhibitory effect on the phosphor FAK^tyr397^ levels (Figs. 1F and S3). These results demonstrated that FC-11 can rapidly and efficiently degrade FAK in the reproductive tissues of male mice. In addition, the location and expression of FAK in the testis were detected by immunofluorescent. Immunostaining revealed that FAK was mainly localized to the basal compartment of seminiferous tubules, which was consistent with previously published data (Siu et al., 2009) (Fig. S4). As above, FC-11 treatment significantly decreased the cytoplasmic expression of FAK, while PF562271 treatment had no effect on FAK levels, again demonstrating the totally different mechanisms of action of the FAK-PROTAC protein degrader FC-11 and the FAK inhibitor PF562271.

Whether FAK protein could be recovered to normal levels after withdrawal of treatment? The mice were raised normally for 2, 4, 6, 9, and 14 days, after withdrawing drug treatment (Fig. 1E). FAK levels recovered gradually over time after FC-11 withdrawal. Except in the preputial gland, the level of FAK in mouse reproductive organs (testis, epididymis and seminal vesicle) was almost normal at 14 days after FC-11 withdrawal. FAK levels in the preputial gland only recovered about 40% in 14 days (Fig. 1G). The above results indicate that FC-11 can achieve reversible regulation of FAK in mice.

Based on the above results, FAK-PROTAC showed a potent and reversible FAK degradation in mouse reproductive tissues of male mice, which indicated that FC-11 can be used as a biological tool for FAK knockdown *in vivo*. Next, the possible functional consequence differences between FAK PROTAC and FAK inhibitor in mouse reproductive system of male mice were further observed. We administered FC-11 (20 mg/kg, BID), PF562271 (10 mg/kg, BID), and vehicle control to 10 week old male C57BL/6N mice via intraperitoneal injections for 13 days. After 13 days treatment, all FC-11 treated males exhibited a significant reduction in the weight or size of the testis, epididymis, seminal vesicle, and preputial gland of 24.2%, 37.5%, 51.6% and 90% of vehicle control, respectively. No reduction was observed in PF562271-treated mice compared to the vehicle-treated group (Figs. 2A and S5). In addition, the number of viable sperm from the caudal epididymis was markedly decreased in FC-11 treated mice. Analysis of sperm motility in FC-11 treated males also revealed a significant (more than five fold) reduction compared to vehicle males. However, there was no significant decrease in sperm viability in PF562271 treated mice compared to vehicle control mice (Fig. 2B and 2C).

**Figure 2:**
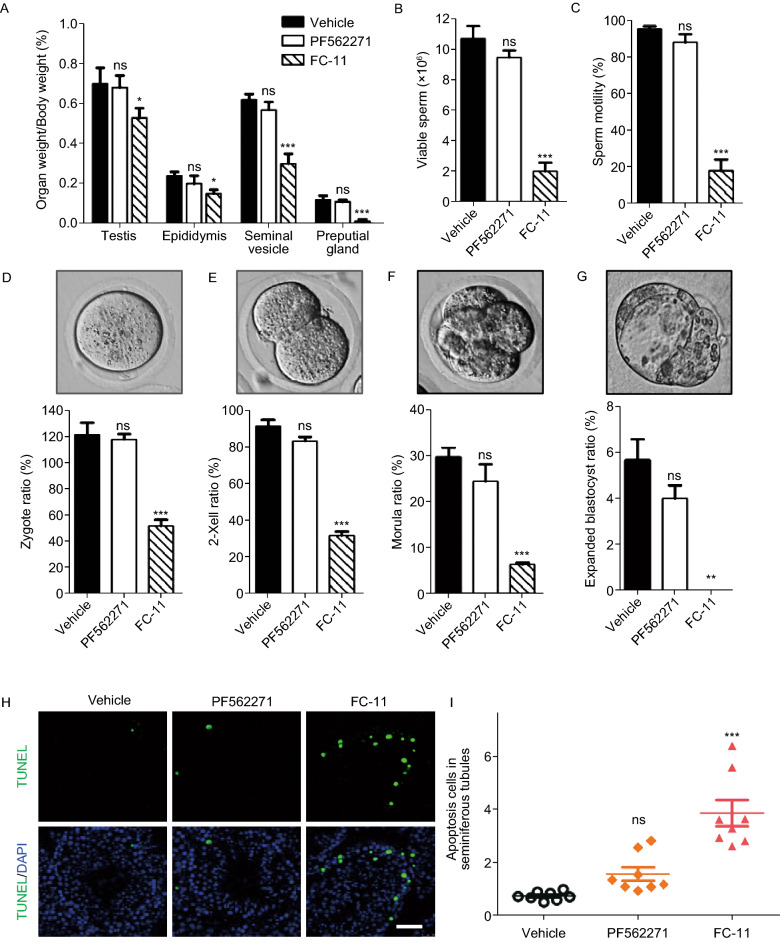
**FAK PROTAC and FAK inhibitor showed different effects on reproductive tissues and sperm phenotypes in male mice.** (A) Organ index of each group mice (Organ index % = organ weight/body weight × 100, *n* = 6). (B and C) Characteristics of sperm phenotype (viable sperms and sperm motility) from each group (*n* = 6). (D–G) Statistical analysis of *in vitro* fertilization rate of spermatozoa and the development of pre-implantation mouse embryos in each group mice, the image represents the morphology of pre-implantation mouse embryos in various developmental stages (*n* = 6). (H) TUNEL staining of testis sections from each group mice (bar, 50 μm). (I) Statistic data of apoptosis cells in seminiferous tubules of testis by TUNEL staining, about 160 seminiferous tubules are selected for statistic in each group. The graphs depict mean ± SD of six mice, ns: no significant, ^*^P < 0.05, ^**^P < 0.01, ^***^P < 0.001 vs. vehicle group

In addition, the fertilization rate and the development of 2-cell, morula, and expanded blastocyst embryos from FC-11 treated mouse sperm was strongly impaired compared to embryos fertilized by sperm from vehicle mice, while the fertilization rate and embryos fertilized by sperm from PF562271-treated mice displayed normal or only slightly impaired embryonic development *in vitro* (Figs. 2D–G and S6). The observed decrease in sperm fertility and impaired embryo development in FC-11 treated males imply that FC-11 has the potential to modulate the fertility of male mice. Furthermore, histopathology and apoptosis analysis of the seminiferous tubules of the testis showed that FC-11, but not PF562271, induced a significant increase in apoptosis of germ cells close to the base membrane of seminiferous tubules compared to vehicle (Figs. 2H, 2I and S7).

In summary, we have described developing a FAK-targeting PROTAC probe for chemical biology study of related non-enzymatic function of FAK in murine reproductive system. This novel strategy has an advantage over current FAK small molecule inhibitors because inhibitors are only applicable to the study of enzymatic functions, not the study of both enzymatic and non-enzymatic function. In this study, the result showed that FAK can be degraded by more than 90% after representative degarder FC-11 treatment, and that it can be recovered to normal levels within two weeks after withdrawing treatment *in vivo*. In contrast to FAK inhibitor PF562271-treated mice, which exhibit an intact reproductive system, FC-11-treated FAK knockdown mice exhibit low sperm viability and motility, and subsequent decreased fertility and poor embryo development due to the impairment of non-enzymatic FAK functions.

Furthermore, in order to make better use of the FAK PROTAC tool in mice, we also detected the general distribution of FC-11 in mice with 20 mg/kg of FC-11 (BID) in 10 week old male C57BL/6N mice through intraperitoneal injections for 5 days. The result displayed that FC-11 can not penetrate the blood–brain barrier, and we did not detect the FAK knockdown in brain of mice. However, FC-11 can work in other mice organs such as liver, spleen, lung, and kindey with different degradation degree, which may further broaden the potential application of FC-11 in other biological studies. In addition, from the celluar FAK selectivity analysis, we also found that FC-11 did not degrade the off-targets of PF562271 such as CDK1, CDK2, CDK7 and FLT3 (Roberts et al., 2008), but FC-11 showed a very slight protein degradation in proline-rich tyrosine kinase 2 (Pyk2) in SRD15 cell line, the homolog of FAK, which have a highly consistent in structure with FAK (45% homology with FAK in amino acid sequence and 61% homology in catalytic domain) (Fig. S8) (Zheng et al., 1998).

Overall, our findings indicate that PROTACs can be used as chemical knockdown tools to study the non-enzymatic functions of proteins, shedding the constraints of traditional small molecule inhibitors. Unlike DNA- or RNA-based protein knockout technology, the PROTAC strategy knocks down target proteins directly, rather than acting on the genome or nucleic acid level, and is suitable for the functional study of embryonic-lethal proteins in adult organisms. Finally, PROTAC probes also provide exquisite temporal control, allowing the knockdown of a target protein of interest at specific developmental time points and enabling the recovery of the target protein after withdrawal of drug treatment.

## Electronic supplementary material

Below is the link to the electronic supplementary material.

Supplementary file1 (PDF 1397 kb)
