# Supplementary material for: FAK-targeting PROTAC as a chemical tool for the investigation of non-enzymatic FAK function in mice
Source: Protein Cell. 2020 May 25;11(7):534–9. doi: 10.1007/s13238-020-00732-8 (PMC7305269; doi:10.1007/s13238-020-00732-8)
Supplement: Supplementary file 1 — Supplementary file1 (PDF 1397 kb) [file 13238_2020_732_MOESM1_ESM.pdf]

SUPPLEMENTARY INFORMATION

FAK-targeting PROTAC as a chemical tool for the investigation of non-enzymatic FAK function in mice

Hongying Gao‡[1,2], Chunwei Zheng‡[3], Jian Du[4], Yue Wu[1], Yonghui Sun[1], Chunsheng Han*[3,5], Kehkooi Kee*[4], and Yu Rao*[1]

[1] MOE Key Laboratory of Protein Sciences, School of Pharmaceutical Sciences, MOE Key Laboratory of Bioorganic Phosphorus Chemistry & Chemical Biology, Tsinghua University, Beijing 100084, P.R. China.

[2] Tsinghua University-Peking University Joint Center for Life Sciences, Beijing 100084, P.R. China.

[3] State Key Laboratory of Stem Cell and Reproductive Biology, Institute of Zoology, Chinese Academy of Sciences, Beijing, 100084, P.R. China.

[4] Center for Stem Cell Biology and Regenerative Medicine, Department of Basic Medical Sciences, School of Medicine, Tsinghua University, Beijing 100084, P.R. China.

[5] University of Chinese Academy of Sciences, Savaid Medical School, Beijing 100049, China

‡ These authors contributed equally to this work.

* Correspondence: hancs@ioz.ac.cn (C. Han), kkee@tsinghua.edu.cn (K. Kee), yrao@tsinghua.edu.cn (Y. Rao)

SUPPLEMENTARY MATERIALS AND METHODS

Synthesis and spectroscopic characterization of compound FC-11

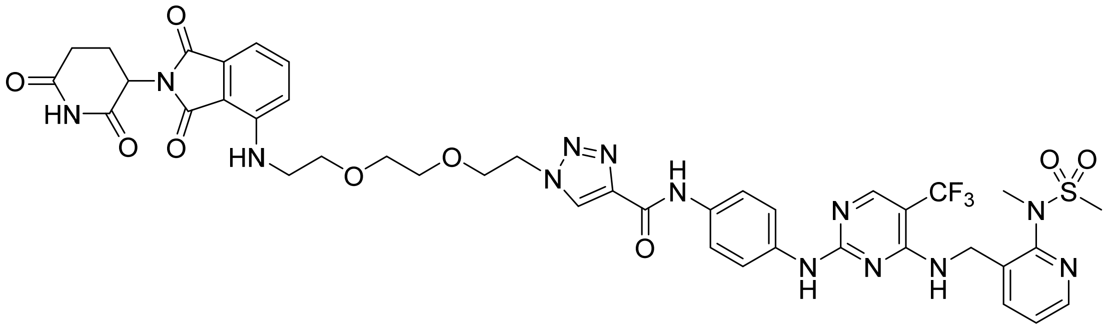

1-(2-(2-(2-((2-(2,6-dioxopiperidin-3-yl)-1,3-dioxoisoindolin-4-yl)amino)ethoxy)ethoxy)ethyl)-*N*-(4-((4-(((2-(*N*-methylmethylsulfonamido)pyridin-3-yl)methyl)amino)-5-(trifluoromethyl)pyrimidin-2-yl)amino)phenyl)-1*H*-1,2,3-triazole-4-carboxamide **(FC-11)**

Synthesis procedure: click reaction route to triazole core. To a stirred solution of the corresponding alkyne (1 eq) and the corresponding azide (1 eq) in *t*-BuOH were added $CuSO_4$ (0.5 eq), VcNa (3 eq) and water (10% to *t*-BuOH). The mixture was stirred at 70 ºC under argon for 8 h. Then the mixture was dissolved in EA and washed with brine, dried over Na_2SO_4, filtered and concentrated. The crude product was purified by column chromatography (DCM:MeOH = 40:1), yielding the corresponding triazole (I.Y. = 40% - 80%).

^{1}H-NMR(400MHz, $CDCl_3$, ppm): 10.05(s, 1H), 8.85(s, 1H), 8.40(d, J = 4.12 Hz, 2H), 8.17(s, 1H), 7.87(s, 1H), 7.79(d, J = 7.16 Hz, 1H), 7.52-7.41(m, 5H), 7.26-7.23(m, 1H), 7.07(d, J = 7.08 Hz, 1H), 6.89(d, J = 8.56 Hz, 1H), 6.52(t, J = 5.12 Hz, 1H), 6.08(s, 1H), 4.96-4.93(m, 3H), 4.60(q, J = 4.52 Hz, 2H), 3.92(t, J = 4.92 Hz, 2H), 3.72(t, J = 5.08 Hz, 2H), 3.64(s, 4H), 3.45(dd, J = 5.56 Hz, J = 10.48 Hz, 2H), 3.27(s, 3H), 3.06(s, 3H), 2.88-2.73(m, 3H), 2.13-2.10(m, 1H).

^{13}C-NMR(100MHz, $CDCl_3$, ppm): 172.91, 169.72, 169.45, 167.74, 160.60, 158.92, 157.85, 154.48, 152.77, 148.21, 146.73, 143.29, 139.19, 136.05, 135.51, 133.88, 132.93, 132.52, 127.18, 126.13, 124.37, 123.45, 120.73, 120.38, 116.83, 111.62, 110.30, 70.59, 70.53, 69.27, 50.71, 48.95, 42.30, 40.59, 37.64, 35.51, 31.95, 31.53, 29.72, 29.39, 22.89, 22.72, 14.16. LC-MS: calculated for $C_{41}H_{43}F_3N_{13}O_9S$ $[M+H]^+$: 950.29, found 950.77.

Sertoli cell and germ cell cultures. C57BL/6N mice at 6 days of age were used for primary undifferentiated germ cells and Sertoli cells isolation. The method was as previously described, with minor modifications (Kanatsu-Shinohara et al., 2003; Kokkinaki et al., 2009; Wang et al., 2015). Briefly, testis were decapsulated with tweezers under the dissection microscope, and then seminiferous tubules were detached gently and washed with DPBS 3 times. The tubules were digested with 1 mg/mL collagenase IV for 5 min at 37°C, then washed 3 times with F12-DMEM and further digested with 0.25% trypsin for 5 min at 37°C. The digested product, containing primarily Sertoli cells and undifferentiated germ cells, was seed in F12-DMEM with 10% FBS and subjected to gravity sedimentation for 2 h. Then the somatic cells attached to the dish and formed patches, while the undifferentiated germ cells loosely attached on the wells. After collection supernatant containing germ cells, non-adherent cells were cultured in F12-DMEM with 20% KSR (KnockOut Serum Replacement) and 100 U/mL penicillin/ streptomycin.

Antibodies. Antibodies used in this study are: FAK (3285) and CDK2 (78B2) were obtained from Cell Signaling Technology (MA, USA); p-FAK (2D11, sc-81493) and CDK7 (C-4, sc-7344) were purchased from Santa Cruz Biotechnology (CA, USA); FLT3 (A7897) was purchased from ABclonal (MA, USA); PYK2 (YE353, ab32571), CDK1 (EPR165, ab133327) and GAPDH (ab8226) were purchased from Abcam

(Cambridge, MA, USA); *β*-tubulin (KM9003) was obtained from Shanghai Sungene Biotech Co., Ltd. (Shanghai, China). Alexa Fluor® 488 donkey anti-rabbit IgG (R37118, green fluorescence, 1:500) was purchased from Thermo Fisher Scientific (Cambridge, MA, USA). FITC-phalloidin (40735ES75) was purchased from yeasen (Shanghai, China). TUNEL (G3250) was purchased from promega (Madison, Wisconsin, USA).

Animals. The experiment animals (C57BL/6N, 10 weeks of age) used in this study were approved by the Tsinghua University Institutional Animal Care and Use Committee (protocol number 18-RY1). Mice were housed in a SPF (specific pathogen-free) unit on a 12 h light/dark cycle (lights off at 19:00) with the ambient temperature is 24 ± 2°C and the humidity is 50 ± 5%, free access to food and water. Six mice were raised in one polyacrylic cage and all the animals were housed for one week under controlled conditions before the experiments. During the period of the study, the care and use of animals were conducted in accordance with the regulations of the Association for Assessment and Accreditation of Laboratory Animal Care (AAALAC).

After drug administration, the mice were euthanized by CO_2 asphyxiation using slow (20%-30%/min) displacement of chamber air with compressed CO_2 at specified time points. Testis, epididymis, seminal vesicle and preputial gland in male mice were removed immediately. The whole testis was fixed with 4% paraformaldehyde (Santa Cruz Biotechnologies, sc-281692), then embedded in paraffin for hematoxylin and eosin staining (H&E) and immunofluorescence (IF) analysis. Other tissues were snapped frozen in liquid nitrogen for western blot analysis. Each time point had at least 3 mice, including controls.

FAK knockdown and recovery *in vivo*. FC-11 was dissolved in DMSO at 40 mg/mL, diluted with Cremophor-EL (C5135, Sigma) and PBS solution in the ratio of 1:2:17 (Drug:Cremophor-EL:PBS) to the work dose of 2 mg/mL. PF562271 was dissolved in DMSO at 20 mg/mL, diluted with the same solvent as FC-11 to 1 mg/mL. The vehicle was DMSO dissolved with Cremophor-EL and PBS in the ratio of 1:2:17. The drug mixture was injected into mice through intraperitoneal (i.p.) injection with the volume of 10 mL/kg, twice a day (BID). At the end of the treatment, take the related tissues immediately and snap frozen in liquid nitrogen for Western blot analysis. All the mice in these studies were weighed daily before injections and fed ad libitum.

For the recovery studies, the mice were treated as the above description for 5 days, then stopped the drug treatment, and removed the testis, epididymis, seminal vesicle and preputial gland immediately at the indicated days (0, 2, 4, 6, 9 and 14 days post-drug treatment), then snap frozen in liquid nitrogen for FAK protein levels analysis.

Organ indexes, viable sperm counts, sperm motility. The mice were sacrificed after drug treatment through CO_2 asphyxiation (20%-30%/min). Testis, epididymis, preputial gland and seminal vesicle were removed immediately and weighed individually to calculate the organ index (organ index % = organ weight/body weight × 100).

Caudal epididymis spermatozoa from one male were prepared in 1 mL of prewarmed Quinn’s Advantage® Fertilizationmedia, and incubated at 37°C for 30 min in a 5% CO_2 incubator prior to sperm number counting and sperm motility analysis. After incubation, sperm samples were diluted with the ratio of 1:100 in 1 mL warmed HTF media (contained with 90% Trypan blue for viable cells staining, Gibco,

15250061) and spotted onto a glass slide, covered with a 22 × 22 mm cover slip. When the sperm settled down after about 2 min, sperms were counted under the differential interference contrast (DIC) optics with a 20 objective.

Sperm motility percentages and velocities (average path velocity) analyzed with Hamilton Thorne's CEROS II system (Ceros, Hamilton-Thorne, Beverly, MA). The procedure was repeated three times for each sample and averaged as described previously(Mortimer et al., 1997; Roy et al., 2007).

Hematoxylin and eosin staining. Tissues embedded in paraffin, mounted on slides and stained with H&E. Tissue sections (5 μm) were rehydrated by passing through xylene (twice, 10 min per time), decreasing gradient concentration of ethanol (100%, 100%, 90%, 80%, 70%, 50% one time, 5 min respectively) and Milli-Q water (3 times, 10 min per time), respectively. After rehydration, sections were stained with hematoxylin (Amresco, 0701) for 1-2 min, washed with tap water, and then continue stained with eosin (Amresco, 0109). Next, sections were dehydrated with increasing gradient concentration of ethanol (70%, 80%, 90%, 100%, 100%) and xylene. Finally the sections were mounted with neutral balsam (Macklin, 822941) for pathological analysis.

Immunofluorescence assays. Paraffin sections were rehydrated as described in H&E stainning. After rehydration, sections were permeabilized with 0.5% Triton X-100 PBS solution for 10 min, washed with PBS solution for 3 times, 10 min per time. Then antigen retrieval was performed by heating sections in 10 mM citrate buffer (pH 6.0) in a microwave for 10 minutes. Sections were allowed to cool and washed in PBS solution. Then sections were blocked with 10% fetal bovine serum (FBS) in PBS for 2 hour. After blocking, sections were incubated with primary antibody (anti rabbit FAK antibody, 1:500 diluted in 3% FBS PBS solution) at 4°C

overnight. Thereafter, sections were incubated with secondary antibody (Alexa Fluor® 488 donkey anti-rabbit IgG, green fluorescence, 1:500 diluted in PBS) for 2 hour at room temperature, washed with PBST 5 times, 5 min per time. Then sections were incubated with 4',6-diamidino-2-phenylindole (DAPI) for visualization of cell nuclei for 10 min. Finally the sections washed with PBST solution twice and mounted with ProLong Gold antifade mountant. Fluorescence images were captured with a Zeiss laser confocal microscope Observer. Z2 LSM 780 (German, Zeiss).

FITC-phalloidin (40735ES75, Yeasen) and TUNEL (G3250, Promega) were stainned according to the protocol of the product from the manufactory.

In vitro fertilization. In vitro fertilization was performed with some modifications based on previous described(Guan et al., 2014). 10-week-old adult male mice were given daily intraperitoneal injections of FC-11 (20 mg/kg, BID), PF562271 (10 mg/kg, BID), or vehicle control over a 13 day period. After treatment, mice were sacrificed and sperm were immediately taken from the caudal epididymis for IVF. Collect mature sperms from caudal epididymis of FC-11 treated males, and added to the HTF medium droplet to capacitation for 1 h at 37°C, 5% CO_2 incubator. Then, collect the same number of cumulus-intact oocytes from superovulated WT female mice (4-week-old), pooled the oocytes and divided into several groups to the fertilization droplet. Put the capacitated sperm to the fertilization droplet containing the eggs to fertilization at 37°C, 5% CO_2 incubator. After 4 to 6 hours incubation, the eggs were washed to remove unbound sperm and transferred to new fertilization droplets. Fertilization rates were evaluated by recording the number of zygote numbers in each group. The development of embryo were evaluated by recording the number of two-cell embryo at twenty-four hours, the morula at seventy-two hours and the expanded blastocyst at ninety-six hours.

Statistical analysis. All data are presented as the mean ± SD, and repeated at least three times. One-way analysis of variance (ANOVA) followed by Dunnett's multiple comparison test was used as a calculated statistical method for data comparison among different treatment groups with Graphpad Prism Version 6. All statistics are representative of biological replicates ($^{*}p < 0.05$, $^{**}p < 0.01$, $^{***}p < 0.001$, ns: no significant).

REFERENCES

Guan, M., Bogani, D., Marschall, S., Raspa, M., Takeo, T., Nakagata, N., and Fray, M. (2014). In vitro fertilization in mice using the MBCD-GSH protocol. Curr Protoc Mouse Biol 4, 67-83.

Kanatsu-Shinohara, M., Ogonuki, N., Inoue, K., Miki, H., Ogura, A., Toyokuni, S., and Shinohara, T. (2003). Long-term proliferation in culture and germline transmission of mouse male germline stem cells. Biol Reprod 69, 612-616.

Kokkinaki, M., Lee, T.L., He, Z., Jiang, J., Golestaneh, N., Hofmann, M.C., Chan, W.Y., and Dym, M. (2009). The molecular signature of spermatogonial stem/progenitor cells in the 6-day-old mouse testis. Biol Reprod 80, 707-717.

Mortimer, S.T., Schevaert, D., Swan, M.A., and Mortimer, D. (1997). Quantitative observations of flagellar motility of capacitating human spermatozoa. Hum Reprod 12, 1006-1012.

Roy, A., Lin, Y.N., Agno, J.E., DeMayo, F.J., and Matzuk, M.M. (2007). Absence of tektin 4 causes asthenozoospermia and subfertility in male mice. FASEB J 21, 1013-1025.

Wang, S., Wang, X., Wu, Y., and Han, C. (2015). IGF-1R signaling is essential for the proliferation of cultured mouse spermatogonial stem cells by promoting the G2/M progression of the cell cycle. Stem Cells Dev 24, 471-483.

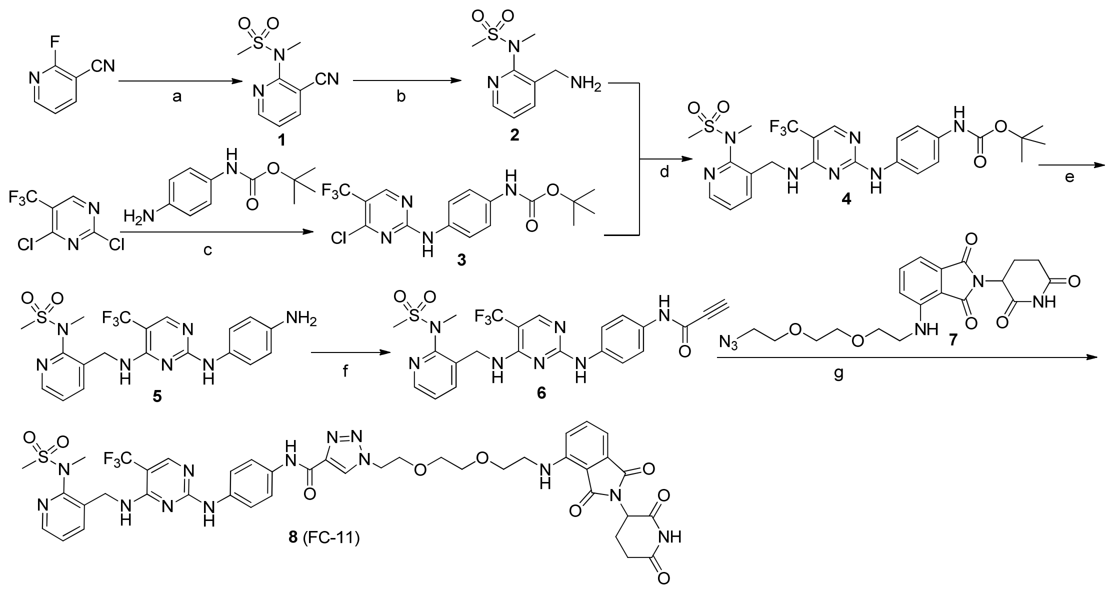

Scheme 1. The optimised synthesis route of FC-11. (a) *t*-BuOK, DMF; (b) Pd/C, H_2, EtOH, EtOAc; (c) $ZnCl_2$, DCM, *t*-BuOH; (d) DIPEA, DCE, *t*-BuOH; (e) TFA, DCM; (f) Propiolic acid, DCC, DMAP, DCM; (g) $CuSO_4$, sodium ascorbate, THF, H_2O.

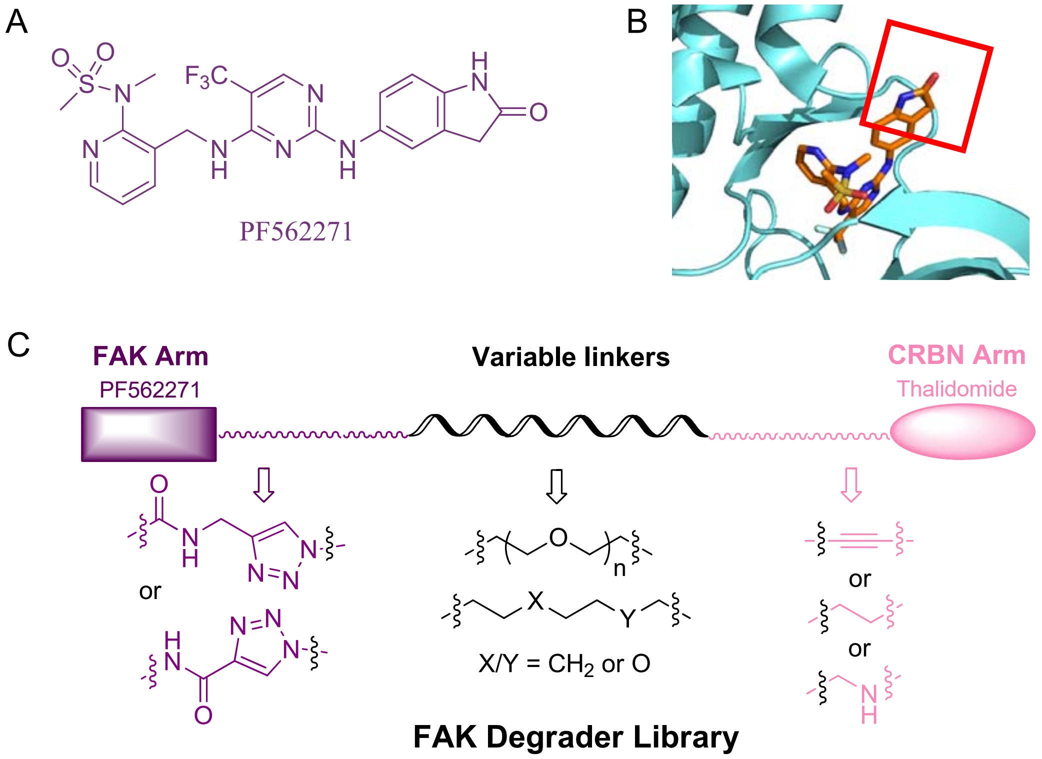

Figure S1. Design of FAK-targeting PROTAC library. (A) Chemical structure of FAK inhibitor PF562271. (B) Binding model of FAK inhibitor PF562271 with FAK protein (PDB: 5TOB). (C) Representative structures of designed FAK PROTAC library.

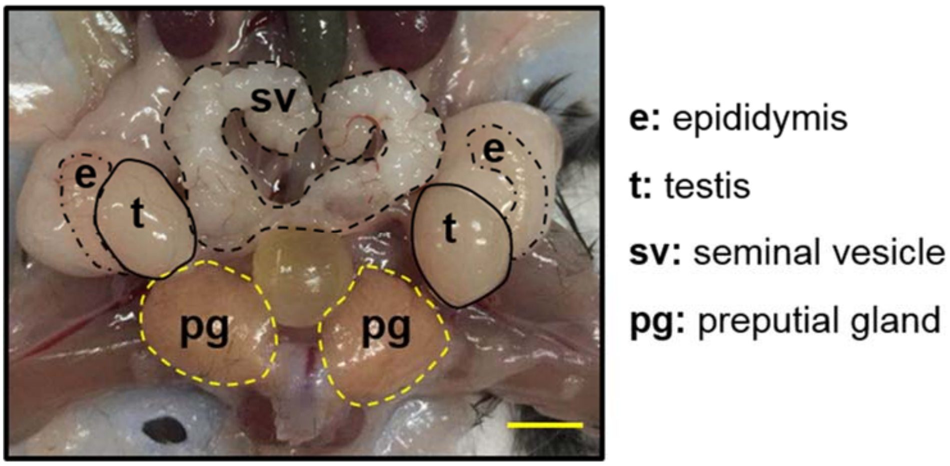

Figure S2. The related organs of mice reproductive system (scale bar, 5 mm).

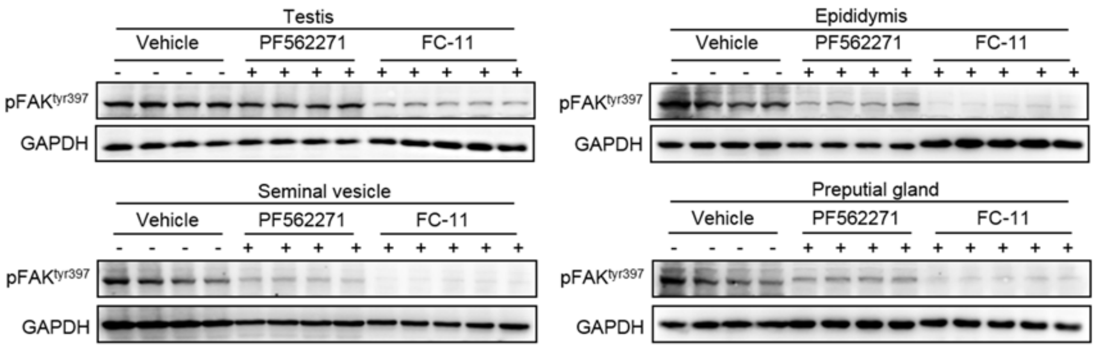

Figure S3. FC-11 leads to more extensive phosphorylation FAK (Tyr 397) downregulation in testis, epididymis, seminal vesicle and preputial gland, respectively. Each lane represented a single mouse (n = 4 or 5).

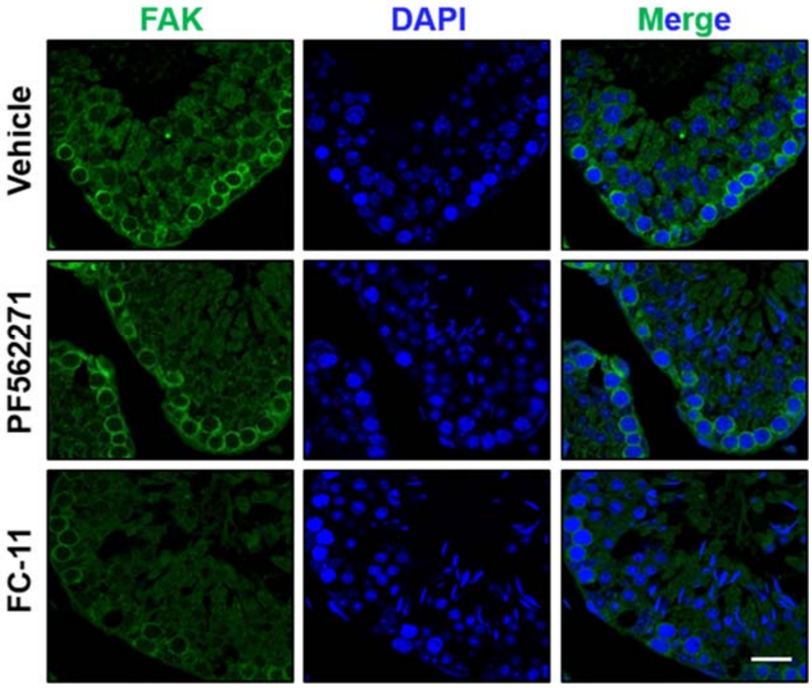

Figure S4. Immunofluorescence indicated FAK protein locations and levels in testis of mice. Cells were counterstained with DAPI (4', 6-diamidino-2-phenylindole) to indicate cell nuclei. Images were acquired utilizing confocal microscopy (Scale bar, 50 μm).

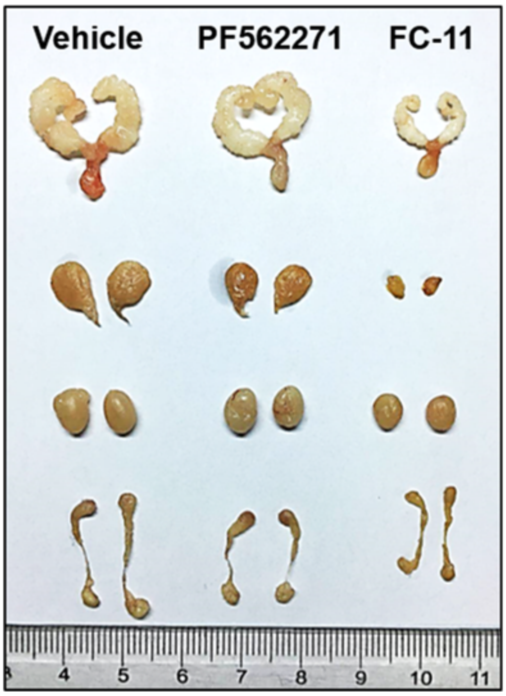

Figure S5. Representative morphology of the testis, epididymis, seminal vesicle and preputial gland in each treated group.

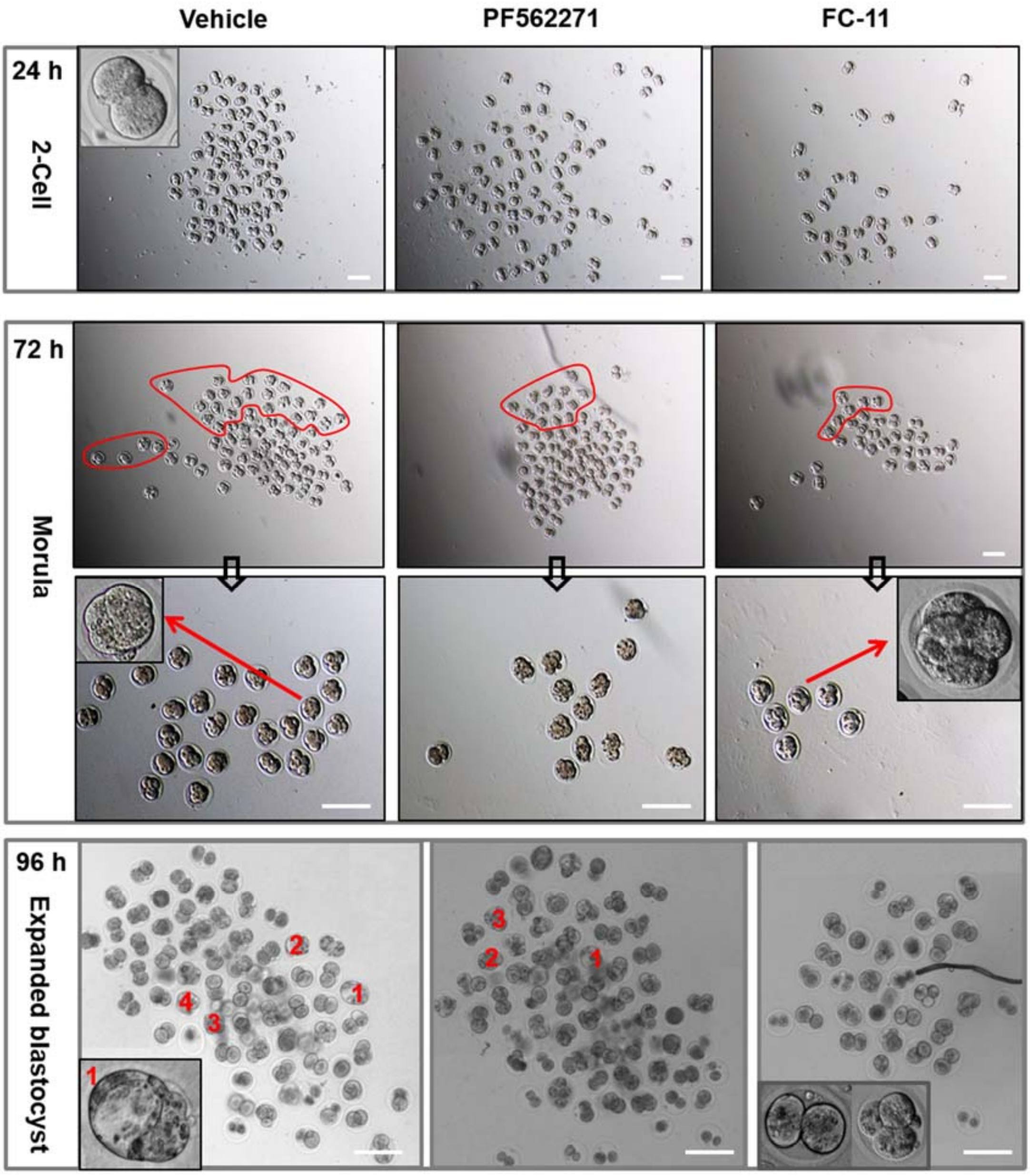

Figure S6. Representative images of the key events during the development of pre-implantation mouse embryos induced by FC-11. 24 h, 72 h and 96 h after in vitro fertilization showing the 2-cell, Morula and Expanded blastocyst embryos, respectively. In the image of 72 h, Morula is putted in the red circle and magnified in an individual image. In the image of 96 h, expanded blastocyst is marked with red numbers (Scale bar, 100 μm).

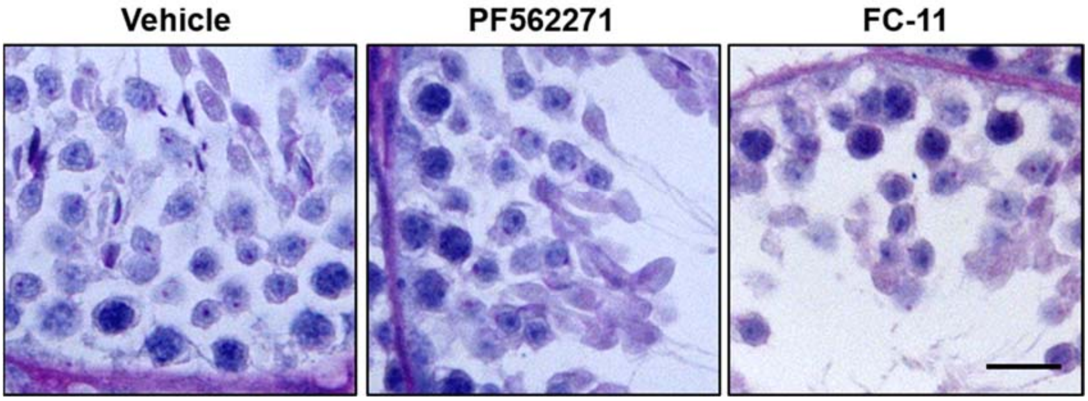

Figure S7. Histological analysis of the anti-spermatogenic effects of FC-11. Scale bars = 200 μm.

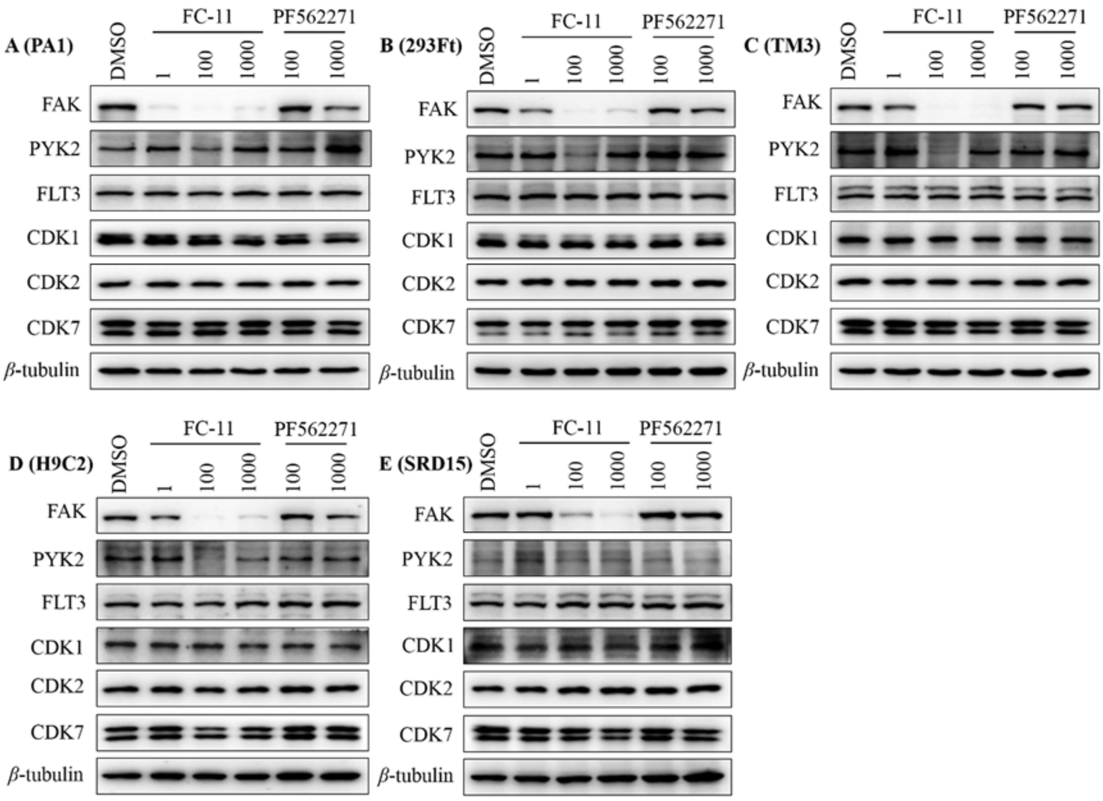

Figure S8. The protein levels of FAK, Pyk2, CDK1, CDK2, CDK7, FLT3 and *β*-tubulin in (A) PA1 (Human ovary cancer cell line), (B) 293Ft (Human embryonic kidney cell line), (C) TM3 (Mouse Leydig cell line), (D) H9C2 (Rat myocardial cell line) and (E) SRD15 (Chinese hamster ovary cell line) cells after 8 h of FC-11 treatment.
